# Baseline Th17/Tc17 and LAG-3 levels serve as candidate exploratory markers for early ixekizumab response in psoriasis

**DOI:** 10.3389/fimmu.2026.1653033

**Published:** 2026-03-27

**Authors:** Nan Yang, Zeyu Chen, Yuxiong Jiang, Jiangluyi Cai, Lian Cui, Yuanyuan Wang, Suyang Lin, Ling Wu, Qian Du, Jun Gu, Yu Gong, Qian Yu, Yuling Shi

**Affiliations:** 1 Department of Dermatology, Shanghai Skin Disease Hospital, Tongji University School of Medicine, Shanghai, China; 2 Institute of Psoriasis, Tongji University School of Medicine, Shanghai, China; 3 Department of Dermatologic Surgery, Shanghai Skin Disease Hospital, Tongji University School of Medicine, Shanghai, China; 4 Department of Dermatology, Shanghai Tenth People’s Hospital, Tongji University School of Medicine, Shanghai, China

**Keywords:** candidate exploratory markers, ixekizumab, LAG-3, psoriasis, Tc17cells, Th17 cells

## Abstract

**Introduction:**

Psoriasis is a T cell-mediated inflammatory skin disease, and biologics have demonstrated promising efficacy. However, some patients remain dissatisfied with early therapeutic outcomes. Immune checkpoint molecules on T cells play critical roles in psoriasis pathogenesis, yet their impact on therapeutic response remains unclear. This study aimed to identify predictive markers for the early therapeutic efficacy of ixekizumab in psoriasis patients.

**Methods:**

Twenty-two patients with plaque psoriasis were enrolled in this study. All participants received ixekizumab for 24 weeks. Peripheral blood was collected at multiple time points, and flow cytometry was used to assess T cell subsets and the expression of immune checkpoint molecules, including T cell immunoreceptor with Ig and ITIM domains (TIGIT), lymphocyte activating gene 3 (LAG-3), cytotoxic T-lymphocyte associated protein 4 (CTLA-4), B and T lymphocyte-associated protein, endothelial protein C receptor (PROCR), podoplanin (PDPN), programmed cell death 1 (PD-1), and B7-Homolog 6 (B7-H6).

**Results:**

Ixekizumab treatment reduced the proportion of T helper 17 (Th17) cells and IL-17-secreting CD8^+^ T (Tc17) cells and downregulated B7-H6 and LAG-3 expression on CD4^+^ T cells by week 24. Similarly, LAG-3 expression on CD8^+^ T cells decreased. Notably, baseline levels of Th17 and Tc17 cells, as well as LAG-3 expression on CD4^+^ and CD8^+^ T cells, were higher in non-early responders than in early responders.

**Conclusion:**

Ixekizumab treatment may restore immune dysregulation in psoriasis patients. Baseline proportions of Th17 and Tc17 cells, along with LAG-3 expression on CD4^+^ and CD8^+^ T cells, may serve as candidate exploratory markers for the early therapeutic efficacy of ixekizumab.

## 1 Introduction

Psoriasis is a chronic, relapsing, immune-mediated skin disease affecting approximately 125 million people globally (1). Plaque psoriasis is the most prevalent, characterized by sharply demarcated erythematous and scaly plaques, often affecting extensor surfaces, intertriginous areas, palms, and soles. Nail involvement may occur in the form of pitting, onycholysis, or oil-drop discoloration. Many patients also suffer from comorbidities, including psoriatic arthritis (PSA), type 2 diabetes, and coronary atherosclerosis, et al (2–4). The pathogenesis involves a feed-forward inflammation loop primarily driven by dysregulated T helper 17 (Th17) and IL-17-secreting CD8^+^ T (Tc17) cells (5). The IL-23/IL-17 axis plays a pivotal role in sustaining keratinocyte activation and amplifying cutaneous inflammation, forming a central pathogenic pathway in psoriasis (6).

Currently, twelve biologics approved by the U.S. Food and Drug Administration (FDA) target tumor necrosis factor (TNF)-⍺ including adalimumab, certolizumab, etanercept, and infliximab, as well as interleukin (IL)-12/IL-23p40 with ustekinumab, IL-17A with ixekizumab and secukinumab, IL-17 receptor with brodalumab, IL-17A and IL-17F with bimekizumab, and IL-23p19 with guselkumab, risankizumab, and tildrakizumab (7, 8). In clinical trials, TNF-α inhibitors achieved 75% improvement in Psoriasis Area and Severity Index (PASI75) response rates ranging from 49% with etanercept at week 12 to 88% with infliximab at week 10, with intermediate rates observed for adalimumab (71% at week 16) and certolizumab (82.6% at week 16) (9–12). Ustekinumab achieved a PASI75 rate of 75.5% at week 12 (13, 14). IL-23 inhibitors exhibited higher PASI75 rates generally showed higher PASI75 responses, including 62% for tildrakizumab at week 12, 91.2% for guselkumab at week 16, and 86.8% for risankizumab at week 12 (15–18). Similarly, IL-17 inhibitors achieved high PASI75 response rates in clinical trials, with 81.6% for secukinumab at week 12, 83.3% for brodalumab at week 12, 89.7% for ixekizumab at week 12, and 76% for bimekizumab as early as week 4 (19–23).

Some biomarkers have been identified as associated with the response to biologics. For TNF-a inhibitors, several single nucleotide polymorphisms (SNPs) have been linked to PASI75 responses at week 12, including rs6661932 (*IVL*), rs2546890 (*IL12B*), rs2145623 (*NFKBIA*), rs9304742 (*ZNF816A*), rs645544 (*SLC9A8*), rs6908425 (*CDKAL1*) and rs4819554 (*IL17A*) (24–27). Additionally, baseline IL-12 serum level is positively correlated with outcomes of etanercept (28). For ustekinumab, four SNPs correlated with treatment outcomes include rs1143623 (*IL1B*), rs1143627 (*IL1B*), rs8177374 (*TIRAP*), and rs5744174 (*TLR5*) (29). HLA-C*06:02-negative patients have been found to be more likely to respond to adalimumab than to ustekinumab (30). Furthermore, enhanced NF-κB p65 phosphorylation in type-2 dendritic cells (DCs) significantly correlates with lack of clinical response to adalimumab at week 12 (31).

Notably, ixekizumab demonstrates high efficacy, with nearly 90% of patients achieving PASI75 by week 12 (22). Real-world studies have further confirmed its rapid onset of action and favorable safety profile (32). Nevertheless, in clinical trials conducted largely in Western populations, approximately 50% of patients attain PASI75 at week 4 (22, 23), indicating variability in early therapeutic response. Some patients experience a delayed onset of action, highlighting the need to identify biomarkers that could predict early clinical responses. However, there are currently no established biomarkers for predicting early responses to ixekizumab.

T-cell development and function rely on three signaling axes: the antigen-specific T cell receptor (TCR), co-stimulatory/co-inhibitory molecules, and cytokine-mediated signals. Co-inhibitory receptors (CIRs) include cytotoxic T-lymphocyte associated protein 4 (CTLA-4), programmed death receptor 1 (PD-1), lymphocyte activating gene 3 (LAG-3), B and T lymphocyte–associated protein (BTLA), T cell immunoreceptor with Ig and ITIM domains (TIGIT), endothelial protein C receptor (PROCR), and podoplanin (PDPN) (33). Additionally, the immunoglobulin superfamily ligand B7-H6 has emerged as a novel regulator of T-cell responses (34–36). Some of these CIRs are critical for the pathogenesis of psoriasis.

LAG-3 is expressed on activated T cells, intrinsically limiting conventional T cell proliferation, expansion and viability (37, 38). In psoriasis patients, LAG-3 is primarily expressed on regulatory T (Treg) cells (39). The proportion of LAG-3^+^ type 1 regulatory T cells (Tr1) in the peripheral blood is reduced compared to healthy individuals and shows a negative correlation with PASI scores (40). In a Phase I randomized study, it was demonstrated that GSK2831781, a high-affinity (sub-nanomolar), humanized IgG1 monoclonal antibody (mAb) targeting LAG-3, effectively reduced disease activity in patients with mild-to-moderate psoriasis (41). TIGIT, another inhibitory receptor expressed on exhausted T cells, Treg cells, and natural killer (NK) cells, is downregulated on circulating CD4^+^ and CD8^+^ T cells in psoriasis and upregulated following infliximab treatment (42, 43), with expression levels negatively correlating with PASI scores (42).

In this study, we evaluated the frequency of circulating Treg, Th17, and Tc17 cells, as well as the expression of immune checkpoint molecules on peripheral T cells during ixekizumab treatment. Our findings suggest that ixekizumab may restore immune dysregulation in psoriasis patients. Furthermore, baseline proportions of Th17 and Tc17 cells, along with LAG-3 expression on CD4^+^ and CD8^+^ T cells, may serve as candidate exploratory markers for the early therapeutic efficacy of ixekizumab.

## 2 Materials and methods

### 2.1 Study design and participants

This was a prospective, Single-arm observational cohort study conducted at Shanghai Skin Disease Hospital. The study was approved by the ethics committees of Shanghai Skin Disease Hospital and conducted in accordance with the Declaration of Helsinki.

**Figure 1:**
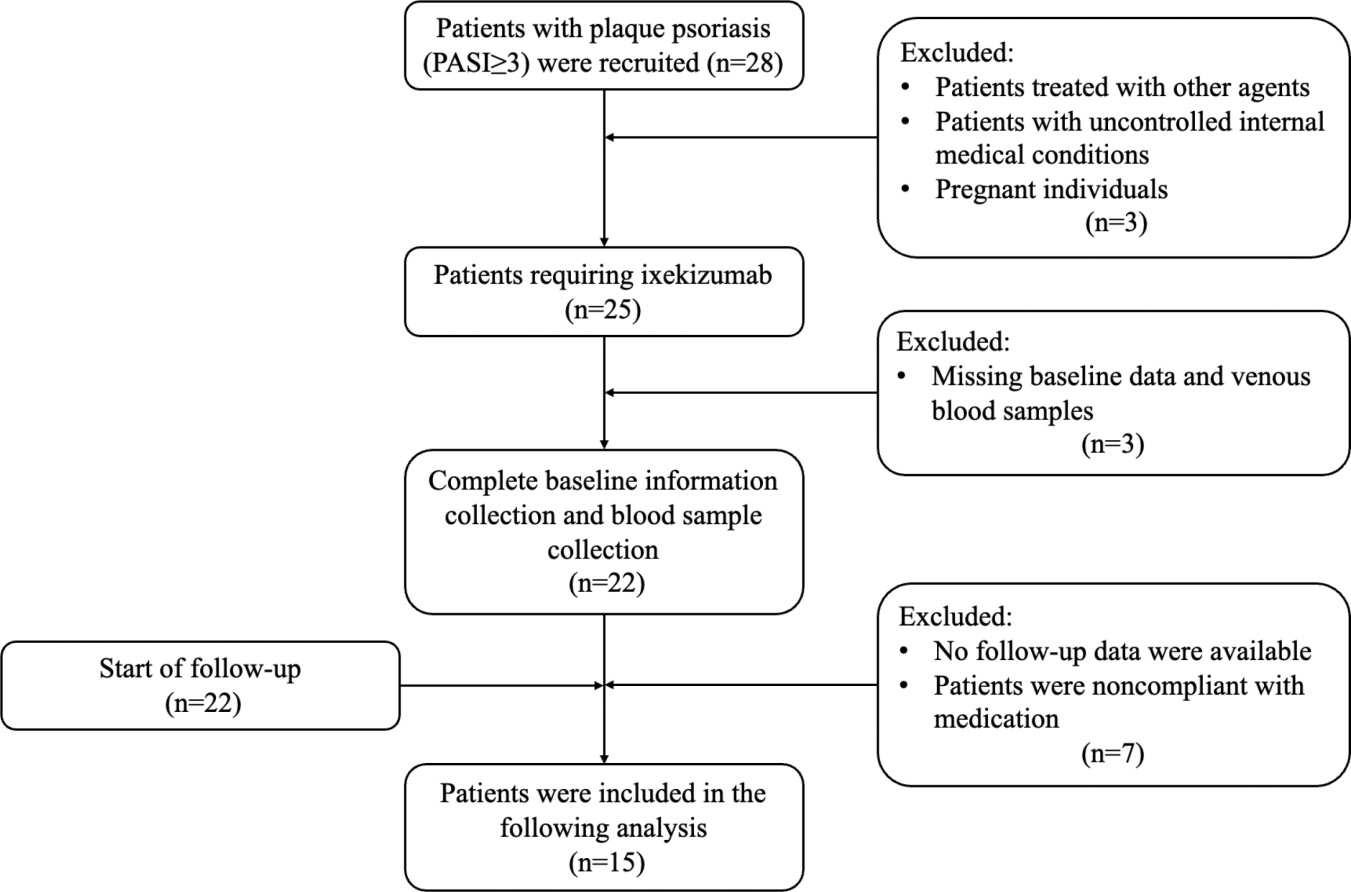
Study flowchart.

The inclusion criteria were adults with a PASI score ≥3 considered to be in need of treatment with biologics. Exclusion criteria were missing baseline data; patients who did not provide follow-up information after screening or who were noncompliant with medication; patients with uncontrolled internal medical conditions, such as diabetes; and pregnant individuals. A total of 22 patients with plaque psoriasis were enrolled for the main analysis (**Figure 1**). All patients received anti–IL-17A monoclonal antibody ixekizumab (Taltz^®^, Eli Lilly and Co.), with an initial dose of 160 mg at week 0, followed by 80 mg at weeks 2, 4, 6, 8, 10, 12, 16, 20, and 24. Venous blood samples were collected from the participants at baseline and at weeks 4, 12, and 24. Written informed consent was obtained from all participants prior to inclusion.

### 2.2 Isolation of peripheral blood mononuclear cells

PBMCs were isolated from patient blood samples using Ficoll-Paque Plus (GE Healthcare, Anaheim, CA), following the manufacturer’s instructions.

### 2.3 Flow cytometric analysis

To assess cytokine secretion, PBMCs were treated *in vitro* with Cell Stimulation Cocktail (#00-4975-03, eBioscience) for 4 hours before staining. Single-cell suspensions were pre-incubated with Fc Receptor Blocking Solution (#422302, BioLegend) for 10 minutes at room temperature. To discriminate dead cells, the cells were stained with Fixable Viability Stain 780 (FVS780, #565388, BD Biosciences) for 15 minutes at room temperature. Subsequently, the cells were stained for 30 minutes with surface marker antibodies in FACS buffer (PBS containing 2% fetal bovine serum) at 4 ˚C. For intracytoplasmic cytokines (ICs) detection, the cells were fixed with IC Fixation Buffer (#00-8222-49, eBioscience) for 30 minutes at 4 ˚C. For intranuclear transcription factor analysis, cells were fixed and permeabilized with Fixation/Permeabilization Diluent and Concentrate (#00–5123-43, eBioscience) for 40 minutes at 4 ˚C. Intracellular antibodies were applied in Permeabilization Buffer (#00-8333-56, eBioscience) for 30 minutes at 4 °C. Data acquisition was performed on a BD FACSCelesta™ Cell Analyzer and analyses were conducted using FLOWJO software (Tree Star, Ashland, or USA). The following human antibodies were used: BV650-conjugated anti-CD3 (clone SK7, #564003, BD Biosciences), BV786-conjugated anti-CD4 (clone SK3, #563881, BD Biosciences), FITC-conjugated anti-CD4 (clone RPA T4, #300506, Biolegend), APC-conjugated anti-CD4 (clone RPA-T4, #300514, eBioscience), AF700-conjugated anti-CD8 (clone SK1, # 564116, Biolegend), BV605-conjugated anti-CD8 (clone SK1, #344723, BD Biosciences), PE-conjugated anti-CD25 (clone BC96, #12-0259-80, eBioscience), APC-conjugated anti-Foxp3 (clone BC96, #17-0529-42, eBioscience), PE-conjugated anti-PROCR (clone RCR- 401, #351904, Biolegend), FITC-conjugated anti-PDPN (clone NC-08, #337026, Biolegend), BV605-conjugated anti-CTLA-4 (clone BNI3, #369610, Biolegend), PerCP-Cy 5.5-conjugated anti-BTLA (clone MIH26, #344514, Biolegend), BV421-conjugated anti-TIGIT (clone A15153G, #372710, Biolegend), AF700-conjugated anti-PD-1 (clone EH12.2H7, #329952, Biolegend), BV510-conjugated anti-LAG-3 (clone 11C3C65, #369318, Biolegend), BV421-conjugated-IL-17A (clone N49-653, #562933, BD Biosciences), PerCP-eFluor 710-conjugated- B7-H6 (clone JAM1EW, #46-6526-42, eBioscience). Gate strategies for T cell subsets are illustrated in **Supplementary Figures S1**–**S3**.

### 2.4 Statistical analysis

Data normality was assessed using the Shapiro–Wilk test. Continuous variables are presented as mean ± standard deviation (SD).

For normally distributed data, comparisons between two independent groups were performed using the two-tailed unpaired Student’s t-test. For non-normally distributed data, the Mann–Whitney U test was applied. Comparisons across more than two groups were conducted using one-way ANOVA (with Bonferroni correction) or the Kruskal–Wallis test followed by Dunn’s multiple comparisons test, as appropriate.

Correlation analyses were performed using Pearson or Spearman correlation coefficients depending on data distribution. Receiver operating characteristic (ROC) curve analysis was used to evaluate the discriminative performance of baseline immunological parameters for early response, and area under the curve (AUC) values with 95% confidence intervals (CI) were calculated. Categorical variables were compared using the chi-square test or Fisher’s exact test when appropriate.

Statistical analyses were performed using GraphPad Prism or SPSS software. Differences were considered statistically significant at *p* < 0.05.

### 2.5 Assessment of treatment effect

The assessment of treatment effect in psoriasis primarily relies on measures such as the PASI and affected body surface area (BSA). In this study, the main efficacy outcome was defined as the PASI50 response rate after 4 weeks of treatment. Additional efficacy outcomes included changed in BSA and DLQI, Physician Global Assessment (PGA)0/1 (‘clear’/’almost clear’) response rates, PASI75, PASI90, PASI100 at weeks 12 and 24. As there is no universally accepted definition of early clinical response in psoriasis, based on existing literature evaluating short-term treatment outcome (44–46), Early Responders (ER) were defined as patients who achieved PASI50 at week 4. Patients who did not achieve PASI50 at week 4 were classified as Non-Early Responders (NER).

## 3 Results

### 3.1 Baseline characteristics and clinical outcomes

Baseline characteristics did not differ significantly between groups, including patients with and without available week 4 PASI data (**Table 1**; **Supplementary Table S1**). The overall cohort had a mean PASI score of 13.2 ± 7.6 and mean BSA of 17.4 ± 12.6%. The mean disease duration was 13.1± 7.7 years.

**Table 1:** Epidemiology of psoriasis patients and controls.

Characteristic	Patients with psoriasis				P value
	Total (n=22)	Early responders (n=7)	Non-early responders (n=8)	Patients without PASI score at week 4 (n=7)	
Male, n (%)	18 (81.8)	6 (85.7)	8 (100)	4 (57.1)	0.095
Age, years, mean (SD)	37.2 (13.2)	35.6 (16.2)	39.6 (15.0)	36 (8.4)	0.539
PASI, mean (SD)	13.2 (7.6)	16.2 (6.3)	10.5 (7.8)	13.4 (9.2)	0.394
BSA, mean (SD)	17.4 (12.6)	17.6 (8.3)	17.3 (12.9)	17.3 (17.3)	0.896
DLQI, mean (SD)	12.7 (6.2)	13.7 (4.7)	12.4 (7.3)	12.3 (6.8)	0.914
PGA, mean (SD)	3.2 (0.8)	3.7 (0.5)	3.0 (0.8)	3.0 (0.8)	0.289
Duration of disease, years, mean (SD)	13.1 (7.7)	10.3 (6.7)	14.1 (8.7)	14.7 (8.0)	0.526

PASI, Psoriasis Area and Severity Index; BSA, body surface area; DLQI, Dermatology Life Quality Index; PGA, Physician’s Global Assessment.

Following ixekizumab treatment, sustained clinical improvement was observed. The mean PASI score decreased to 5.6 ± 3.1 at week 4 and further declined to 2.3 ± 2.8 and 1.5 ± 2.4 at weeks 12 and 24, respectively. Similarly, mean BSA decreased to 10.0 ± 6.2% at week 4, and 4.1 ± 5.2% at week 12, and 2.6 ± 3.7% at week 24. PGA 0/1 response rates increased overtime, reaching 62.5% at week 12, and 72.7% at week 24. The mean percentage improvement in PASI from baseline was 50.5%, 82.4%, and 91.7% at weeks 4, 12, and 24, respectively (**Table 2**). Importantly, the median PASI improvement at week 4 was 49.1%, supporting PASI50 as a clinically reasonable and distribution-based cutoff for defining early response.

**Table 2:** Clinical response to ixekizumab over 24 weeks.

Outcome measure	Baseline (n=22)	Week 4 (n=15)	Week 12 (n=16)	Week 24 (n=11)
PASI, mean (SD)	13.2 (7.6)	5.6 (3.1)	2.3 (2.8)	1.5 (2.4)
PASI Change from baseline, mean (SD)	–	-7.6 (6.1)	-11.8 (6.7)	-12.0 (6.5)
PASI %improvement, mean (SD)	–	50.5 (22.7)	82.4 (12.9)	91.7 (10.0)
PASI response				
≥50% improvement, n (%)	–	7 (46)	16 (100)	11 (100)
≥75% improvement, n (%)	–	2 (13.3)	12 (75.0)	10 (90.1)
≥90% improvement, n (%)	–	0 (0)	4 (25.0)	8 (72.7)
100% improvement, n (%)	–	0 (0)	3 (18.8)	4 (36.3)
BSA, mean (SD)	17.4 (12.6)	10.0 (6.2)	4.1 (5.2)	2.6 (3.7)
BSA Change from baseline, mean (SD)	–	-7.4 (7.8)	-14.23 (12.96)	-15.29 (12.78)
PGA (0/1), n (%)	–	0 (0)	10 (62.5)	8 (72.7)
DLQI, mean (SD)	12.7 (6.2)	7.3 (7.6)	5.7 (5.3)	2.3 (5.3)
DLQI, Change from baseline, mean (SD)	–	-4.8 (9.5)	-6.3 (8.7)	-10.7 (8.5)

PASI, Psoriasis Area and Severity Index; BSA, body surface area; DLQI, Dermatology Life Quality Index; PGA, Physician’s Global Assessment.

### 3.2 Ixekizumab treatment decreased the proportions of Th17 and Tc17 cells

**Figure 2:**
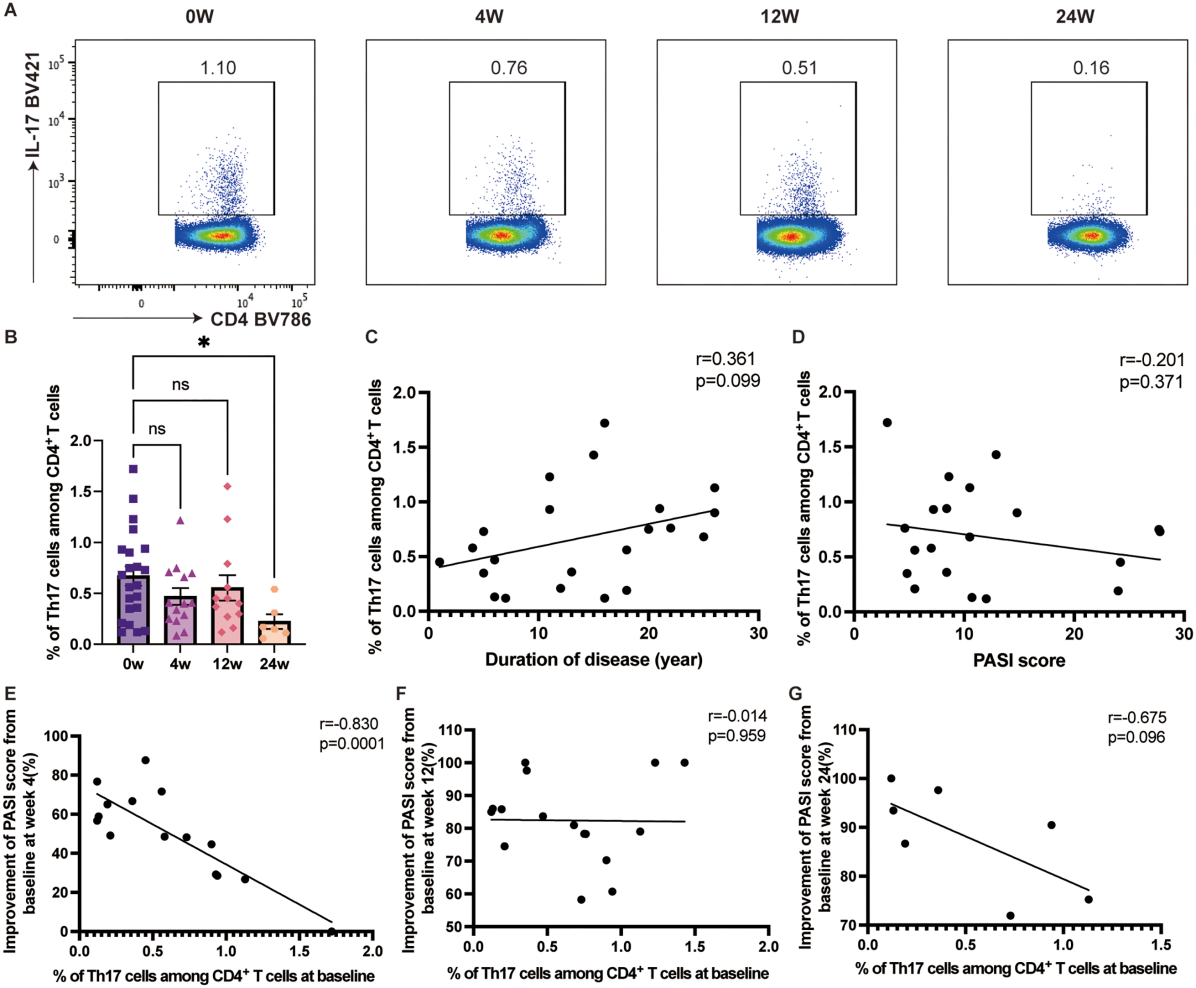
The proportion of Th17 cells in the circulating CD4^+^ T cells. **(A, B)** The proportion of Th17 cells in the circulating CD4^+^ T cells in psoriasis patients treated with ixekizumab at baseline (n=22), week 4 (n=14), week 12 (n=12), and week 24 (n=6). **(C)** The correlation of Th17 cell proportion and the duration of disease of psoriasis patients (n = 22). **(D)** The correlation of Th17 cell proportion in the circulating CD4^+^ T cells and PASI score (n=22). **(E)** The correlation of Th17 cell proportion in the circulating CD4^+^ T cells and improvement of PASI score at week 4 (n=15). **(F)** The correlation of Th17 cell proportion in the circulating CD4^+^ T cells and improvement of PASI score at week 12 (n=17). **(G)** The correlation of Th17 cell proportion in the circulating CD4^+^ T cells and improvement of PASI score at week 24 (n=7). *p < 0.05.

**Figure 3:**
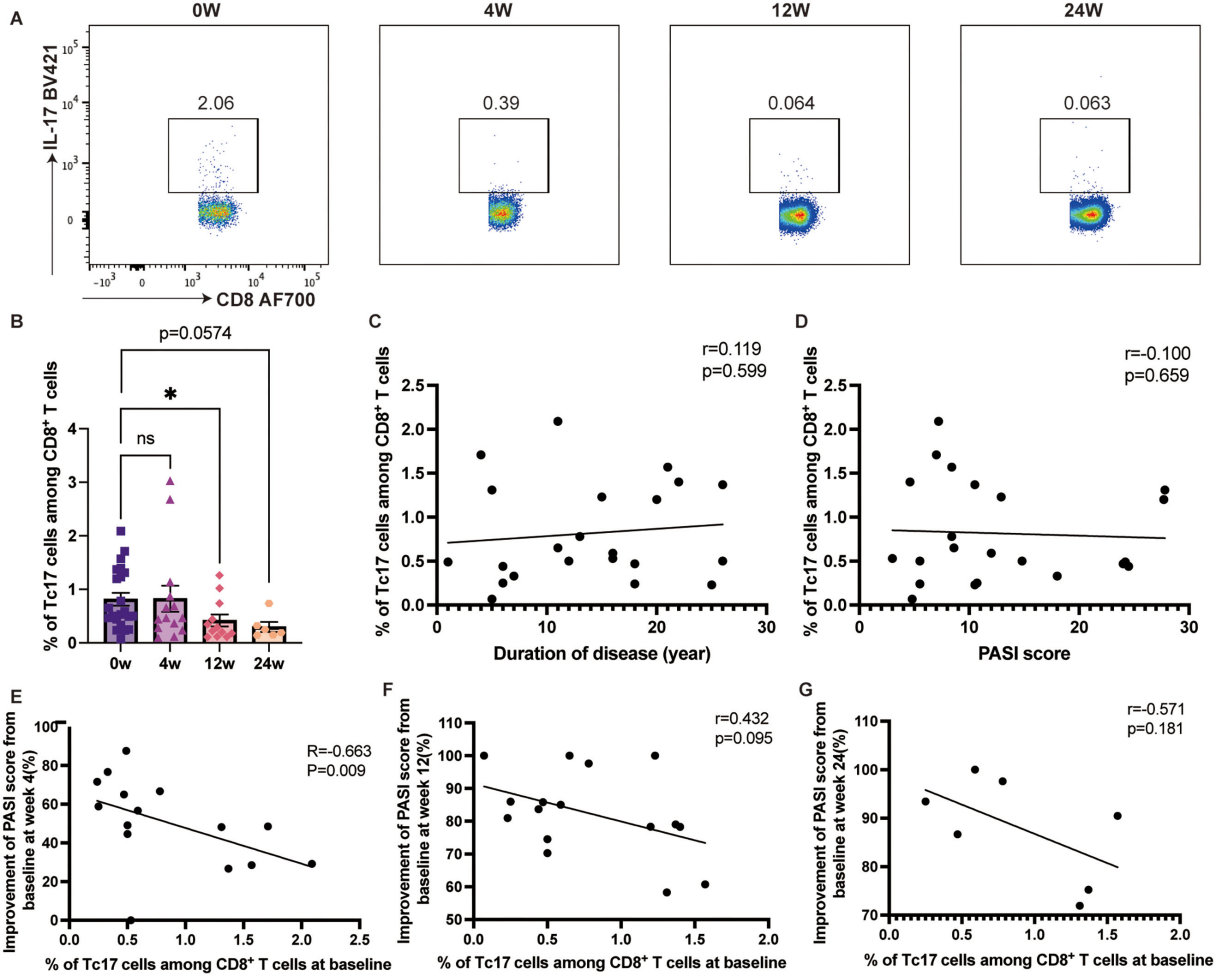
The proportion of Tc17 cells in the circulating CD8^+^ T cells. **(A, B)** IL-17 expression in the circulating CD8^+^ T cells in psoriasis patients treated with ixekizumab at baseline (n=22), week 4 (n=14), week 12 (n=12), and week 24 (n=6). **(C)** The correlation of Tc17 cell proportion and the duration of disease of psoriasis patients (n = 22). **(D)** The correlation of Tc17 cell proportion in the circulating CD8^+^ T cells and PASI score (n=22). **(E)** The correlation of Tc17 cell proportion in the circulating CD8^+^ T cells and improvement of PASI score at week 4 (n=15). **(F)** The correlation of Tc17 cell proportion in the circulating CD8^+^ T cells and improvement of PASI score at week 12 (n=17). **(G)** The correlation of Tc17 cell proportion in the circulating CD8^+^ T cells and improvement of PASI score at week 24 (n=7). *p < 0.05.

Following ixekizumab treatment, the percentage of Th17 cell proportion among circulating CD4^+^ T cells decreased from 0.67% to 0.23% by week 24 (*p=*0.0158) (**Figures 2A, B**). However, no significant correlation was observed between Th17 cell proportion and either disease duration or PASI score (**Figures 2C, D**). Notably, baseline Th17 cell proportion was significantly negatively correlated with PASI score improvement at week 4 (r=-0.8301, 95% CI [-0.9418– -0.5529], p<0.0001) (**Figure 2E**), although no such correlation was found at weeks 12 or 24 (**Figures 2F, G**). Similarly, Tc17 cell proportion in circulating CD8^+^ T cells decreased after ixekizumab treatment, with significant difference (*p=*0.0417) at week 12 (**Figures 3A, B**). Baseline Tc17 cell proportion was significantly negatively correlated with PASI score improvement at week 4 (r=-0.6631, 95% CI [-0.8811– -0.2125], *p=*0.0085), while no significant correlation was found between Tc17 cell proportion and PASI score, disease duration, or therapeutic efficacy at weeks 12 and 24 (**Figures 3C–G**).

Patients receiving ixekizumab did not exhibit any significant alteration in the percentage of Treg cells among peripheral CD4^+^ T cells, nor was this percentage correlated with PASI score, disease duration, or treatment efficacy (**Supplementary Figures S4A–G**). However, the Th17/Treg ratio was significantly reduced by week 24 (*p=*0.0103) (**Supplementary Figure S4H**), but the baseline Th17/Treg ratio was not associated with disease duration or PASI score (**Supplementary Figures S4I, J**). Notably, the baseline Th17/Treg ratio was negatively correlated with PASI score improvement at week 4 (r=-0.6416, 95% CI [-0.8684– -0.1929], *p=*0.0099) (**Supplementary Figure S4K**), but it did not influence therapeutic efficacy at weeks 12 and 24 (**Supplementary Figures S4L, M**).

### 3.3 Ixekizumab treatment decreased LAG-3 expression on both CD4^+^ and CD8^+^ T cells

**Figure 4:**
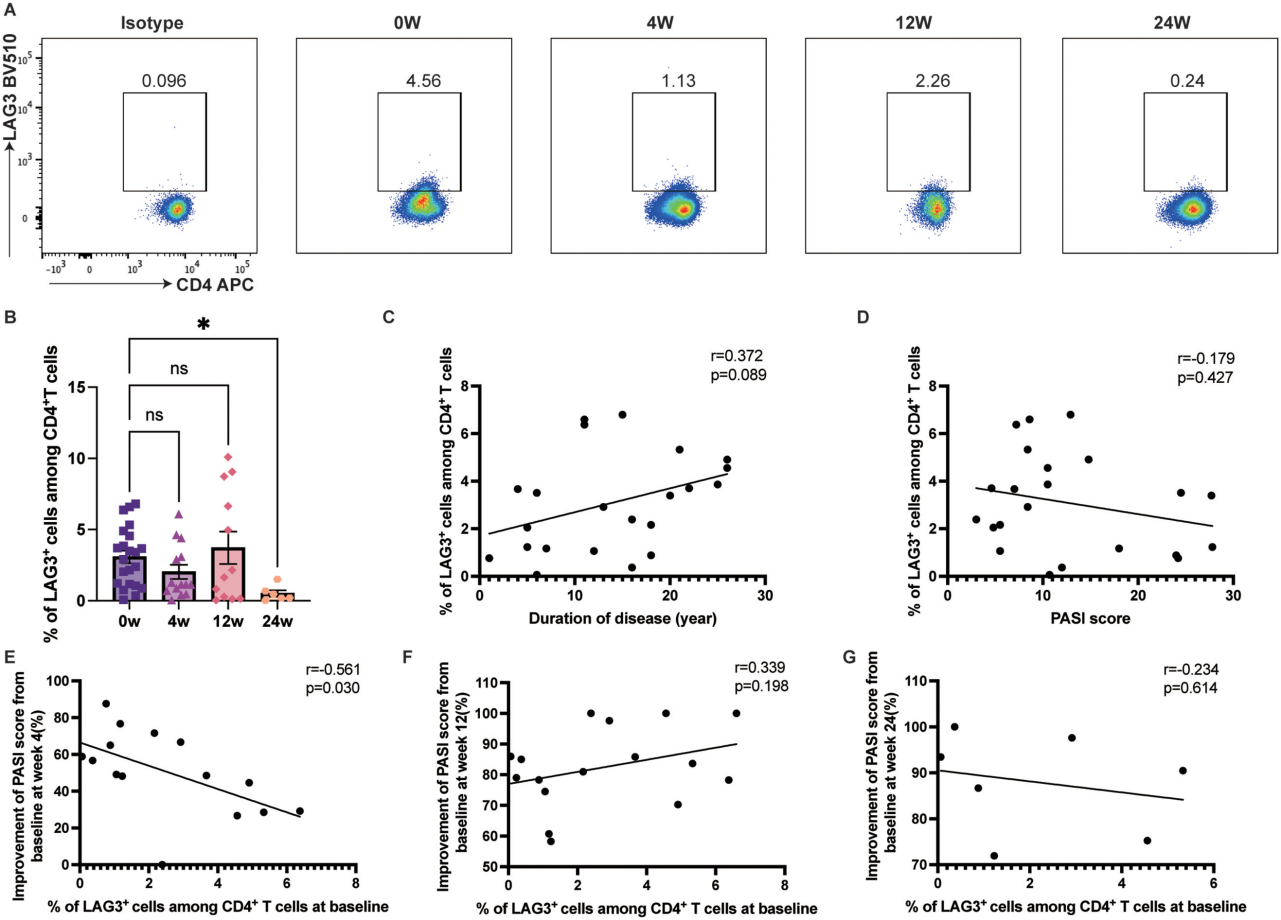
LAG-3 expression on the circulating CD4^+^ T cells. **(A, B)** LAG-3 expression on the circulating CD4^+^ T cells on psoriasis patients treated with ixekizumab at baseline (n=22), week 4 (n=14), week 12 (n=12), and week 24 (n=6). **(C)** The correlation of LAG-3 expression on the circulating CD4^+^ T cells and the duration of disease of psoriasis patients (n = 22). **(D)** The correlation of LAG-3 expression on the circulating CD4^+^ T cells and PASI score (n=22). **(E)** The correlation of the LAG-3 expression and improvement of PASI score at week 4 (n=15). **(F)** The correlation of the LAG-3 expression and improvement of PASI score at week 12 (n=17). **(G)** The correlation of the LAG-3 expression and improvement of PASI score at week 24 (n=7). *p < 0.05.

LAG-3 expression on circulating CD4^+^ T cells decreased from 3.08% to 0.51% at week 24 (*p=*0.0121) (**Figures 4A, B**). While LAG-3 expression on CD4^+^ T cells tended to increase over the disease course (**Figure 4C**), it showed no correlation with PASI scores (**Figure 4D**). Furthermore, baseline LAG-3 expression was negatively correlated with PASI score improvement at week 4 (r=-0.5605, 95% CI [-0.8335– -0.0677], *p=*0.0297) (**Figure 4E**), although it did not influence therapeutic efficacy at weeks 12 and 24 (**Figures 4F, G**).

**Figure 5:**
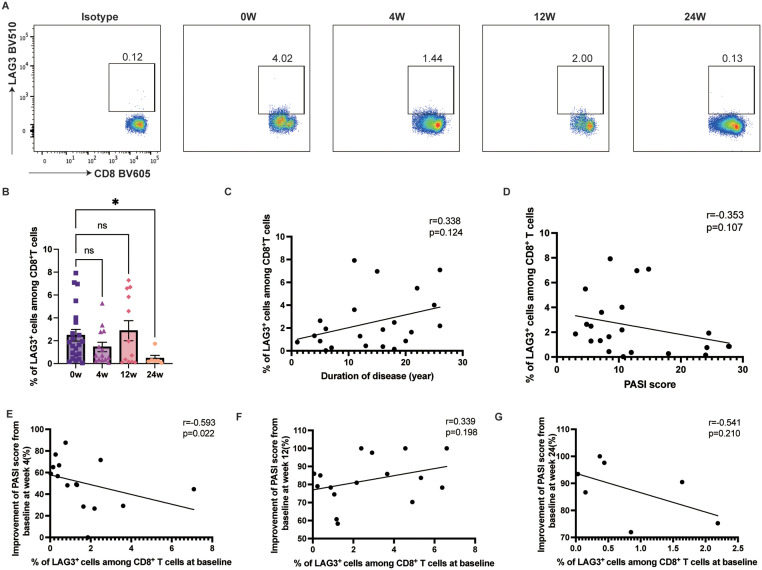
LAG-3 expression on the circulating CD8^+^ T cells. **(A, B)** LAG-3 expression on the circulating CD8^+^ T cells on psoriasis patients treated with ixekizumab at baseline (n=22), week 4 (n=14), week 12 (n=12), and week 24 (n=6). **(C)** The correlation of LAG-3 expression on the circulating CD8^+^ T cells and the duration of disease of psoriasis patients (n = 22). **(D)** The correlation of LAG-3 expression on the circulating CD8^+^ T cells and PASI score (n=22). **(E)** The correlation of the LAG-3 expression and improvement of PASI score at week 4 (n=15). **(F)** The correlation of the LAG-3 expression and improvement of PASI score at week 12 (n=17). **(G)** The correlation of the LAG-3 expression and improvement of PASI score at week 24 (n=7). *p < 0.05.

Similarly, LAG-3 expression on circulating CD8^+^ T cells decreased from 2.48% to 0.47% by week 24 after treatment (*p=*0.0307) (**Figures 5A, B**). However, no significant association was found between LAG-3 expression on circulating CD8^+^ T cells and disease duration or PASI score (**Figures 5C, D**). Baseline LAG-3 expression on circulating CD8^+^ T cells was negatively correlated with PASI score improvement at week 4 (r=-0.5929, 95% CI [-0.8523– -0.0992], *p=*0.0222) (**Figure 5E**), although it did not influence therapeutic efficacy at weeks 12 and 24 (**Figures 5F, G**).

### 3.4 TIGIT expression on CD4^+^T cells was negatively correlated with PASI score improvement at week 4

**Figure 6:**
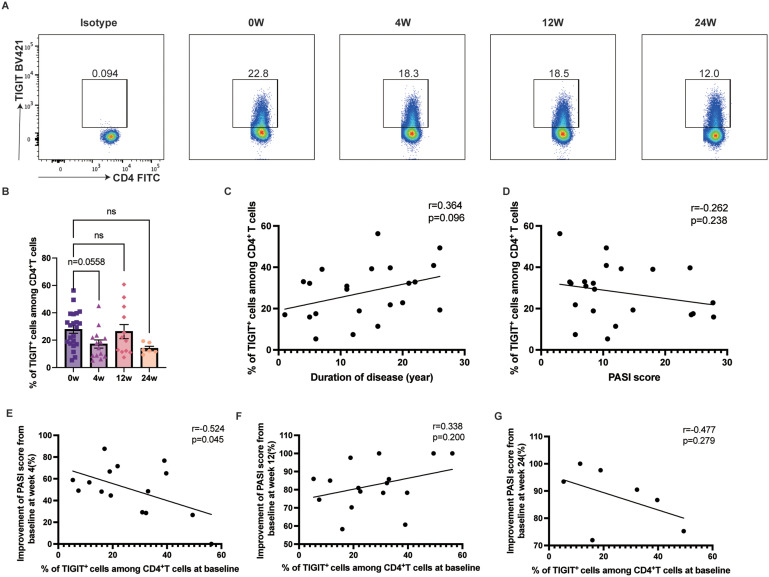
TIGIT expression on the circulating CD4^+^ T cells. **(A, B)** TIGIT expression on the circulating CD4^+^ T cells in psoriasis patients treated with ixekizumab at baseline (n=22), week 4 (n=14), week 12 (n=12), and week 24 (n=6). **(C)** The correlation of TIGIT expression on the circulating CD4^+^ T cells and the duration of disease of psoriasis patients (n = 22). **(D)** The correlation of TIGIT expression in the circulating CD4^+^ T cells and PASI score (n=22). **(E)** The correlation of the TIGIT expression and improvement of PASI score at week 4 (n=15). **(F)** The correlation of the TIGIT expression and improvement of PASI score at week 12 (n=17). **(G)** The correlation of the TIGIT expression and improvement of PASI score at week 24 (n=7).

TIGIT expression on CD4^+^ T cells tended to decrease after ixekizumab treatment, although the changes were not statistically significant (**Figures 6A, B**). Additionally, there appeared to be a positive correlation between TIGIT expression on CD4^+^ T cells and disease duration (**Figure 6C**). However, TIGIT expression on CD4^+^ T cells was not associated with PASI score at baseline (**Figure 6D**). Baseline TIGIT expression on CD4^+^ T cells was negatively associated with early therapeutic efficacy (r=-0.5238, 95% CI [-0.8169– -0.0158], *p=*0.0450), but it did not significantly affect efficacy at weeks 12 and 24 (**Figures 6E–G**). Furthermore, there was no significant change in TIGIT expression on circulating CD8^+^ T cells (**Supplementary Figure S5**).

### 3.5 Ixekizumab treatment decreased B7-H6 expression on CD4^+^T cells

**Figure 7:**
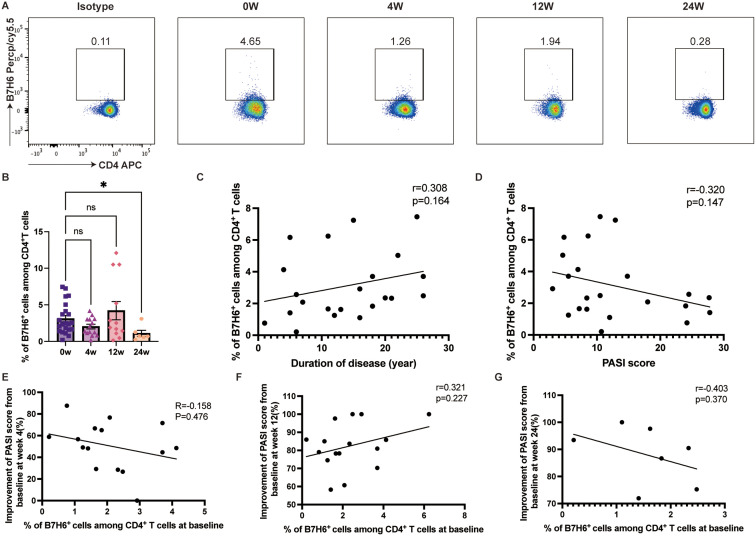
B7H6 expression on the circulating CD4^+^ T cells **(A, B)** B7-H6 expression on the circulating CD4^+^ T cells in psoriasis patients treated with ixekizumab at baseline (n=22), week 4 (n=14), week 12 (n=12), and week 24 (n=6). **(C)** The correlation of B7-H6 expression on the circulating CD4^+^ T cells and the duration of disease of psoriasis patients (n = 22). **(D)** The correlation of B7-H6 expression on the circulating CD4^+^ T cells and PASI score (n=22). **(E)** The correlation of the B7-H6 expression and improvement of PASI score at week 4 (n=15). **(F)** The correlation of the B7-H6 expression and improvement of PASI score at week 12 (n=17). **(G)** The correlation of the B7-H6 expression and improvement of PASI score at week 24 (n=7). *p < 0.05.

B7-H6, a recently identified ligand of the B7 family, has been explored in various solid cancers. By binding to the NK cell receptor NKp30, B7-H6 regulates innate immune responses but may also limit antitumor T-cell responses through NK cell recognition (34, 47). Psoriasis patients exhibited a decrease in B7-H6 expression on circulating CD4^+^ T cells following ixekizumab treatment at week 24 (*p=*0.0424) (**Figures 7A, B**). However, no associations were found between B7-H6 expression and disease duration or PASI score (**Figures 7C, D**). Additionally, B7-H6 expression did not impact therapeutic efficacy (**Figures 7E–G**). A similar change trend was observed in B7H6 expression on CD8^+^ T cells (**Supplementary Figure S6**).

### 3.6 CTLA-4, BTLA, PROCR, PDPN, and PD-1 expression were not significantly altered on circulating T cells of psoriasis patients receiving ixekizumab

CTLA-4, expressed on activated T cells, B cells, and monocyte-derived DCs, inhibits T cell activation by competing with CD28 for binding to B7 family molecules (48). CTLA-4^+^ T cells are more abundant in psoriatic lesional skin compared to healthy skin, with expression levels negatively correlated with disease severity (49). BTLA, a member of the CD28 immunoglobulin superfamily, functions as an inhibitory receptor that suppresses T and B cell activation and cytokine secretion (50, 51). PROCR is expressed in many cell types, including hematopoietic stem cells and tumor cells. Recent studies demonstrated that PROCR also binds other ligands, including TCR present on a subset of Vδ2−γδ T cells (52). PDPN, expressed on keratinocytes and inflammatory cells such as monocytes, promotes IL-17 secretion in inflammatory skin diseases (53, 54). PD-1, expressed on activated and exhausted T cells, inhibits T cell proliferation, polarization, and cytokine production via interaction with PD-Ligand (L)1 and PD-L2 (55). In psoriasis, CD4^+^ and CD8^+^ T cells from PBMCs show decreased PD-1 expression (56, 57). However, there was neither significant alteration of CTLA-4, BTLA, PROCR, PDPN, and PD-1 expression on circulating T cells nor any correlation with the disease severity (**Supplementary Figures S7**-**S11**). Nonetheless, CTLA-4 expression on CD8^+^ T cells was positively correlated with disease duration (**Supplementary Figure 7J**), and BTLA expression on CD4^+^ T cells was negatively correlated with duration of disease duration (**Supplementary Figure S8C**).

### 3.7 Early responders exhibited lower proportions of Th17 and Tc17 cells, along with reduced LAG-3 expression on circulating CD4^+^T and CD8^+^T cells at baseline

**Figure 8:**
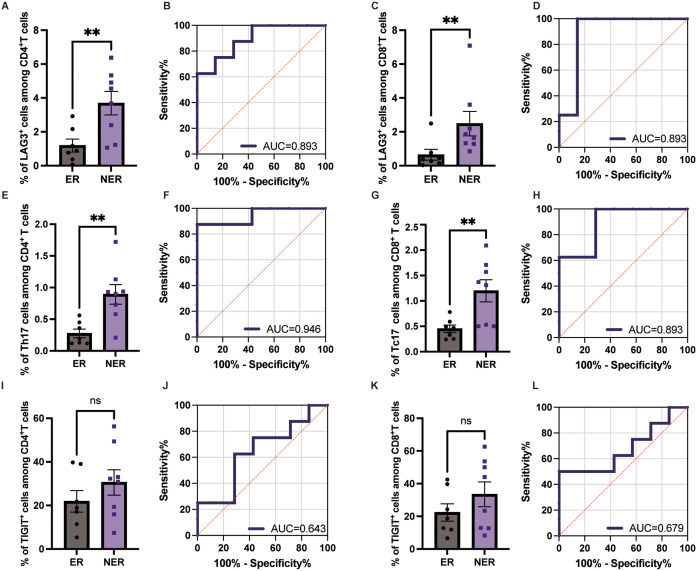
The proportion of Th17 and Tc17 cells, along with LAG-3 and TIGIT expression on circulating T cells, influences early treatment efficacy. **(A)** LAG-3 expression on the circulating CD4^+^ T cells in early responders (n=7) and non early responders (n=8). **(B)** ROC analysis for predicting early efficacy based on LAG-3 expression on the circulating CD4^+^ T cells. **(C)** LAG-3 expression on the circulating CD8^+^ T cells in early responders (n=7) and non early responders (n=8). **(D)** ROC analysis for predicting early efficacy based on LAG-3 expression in the circulating CD8^+^ T cells. **(E)** Th17 cell proportion in the circulating CD4^+^ T cells in early responders (n=7) and non early responders (n=8). **(F)** ROC analysis for predicting early efficacy based on Th17 cell proportion in the circulating CD4^+^ T cells. **(G)** Tc17 cell proportion in the circulating CD8^+^ T cells in early responders (n=7) and non early responders (n=8). **(H)** ROC analysis for predicting early efficacy based on Tc17 cell proportion in the circulating CD8^+^ T cells. **(I)** TIGIT expression on the circulating CD4^+^ T cells in early responders (n=7) and non early responders (n=8). **(J)** ROC analysis for predicting early efficacy based on TIGIT expression on the circulating CD4^+^ T cells. **(K)** TIGIT expression on the circulating CD8^+^ T cells in early responders (n=7) and non early responders (n=8). **(L)** ROC analysis for predicting early efficacy based on TIGIT expression in the circulating CD8^+^ T cells. **p < 0.01. ER, early responders; NER, non early responders.

Given the significant correlations between baseline Th17 and Tc17 proportions, LAG-3 expression, and early PASI score improvement (**Figures 2E**, **4E**, **5E**, **6E**), we investigated whether these markers could distinguish early and non-early responders. The expression of LAG-3 on CD4^+^ (*p=*0.0096) and CD8^+^ T (*p=*0.0093) cells, and the Th17 (*p=*0.0041) and Tc17 cell (*p=*0.0090) proportion were significantly higher in non-early responders than in early responders. (**Figures 8A–H**). No significant differences were observed in TIGIT expression between the groups (**Figures 8I–L**). Furthermore, ROC curves were used to assess the predictive value of immune checkpoint molecule expression for early efficacy. The AUC values for LAG-3 expression on CD4^+^ and CD8^+^ T cells, and the Th17 and Tc17 cell proportion were 0.893 (*p=* 0.011, 95% CI [0.7315–1.000]), 0.893 (*p=* 0.011, 95% CI [0.6899–1.000]), 0.946 (*p=* 0.004, 95% CI [0.8299–1.000]), and 0.893 (*p=* 0.011, 95% CI [0.7249–1.000]), respectively, indicating that these biomarkers may serve as potential predictors of early efficacy for ixekizumab. Additionally, optimal predictive thresholds for LAG-3 expression on CD4^+^ and CD8^+^ T cells, and the Th17 and Tc17 cell proportion were 3.295 (J = 0.625), 0.805 (J = 0.857), 0.570 (J = 0.875), and 0.495 (J = 0.714), respectively (**Figures 8B, D, F, H**).

## 4 Discussion

In this study, we demonstrated that patients with lower baseline levels of Th17 and Tc17 cells and LAG-3 expression were more likely to respond early, suggesting that these biomarkers may help predict early treatment response and guide personalized therapies.

The central role of IL-17 in psoriasis pathogenesis is well-established. Previous studies have demonstrated reduced IL-17 expression in lesional skin following anti–IL-17 therapy (58). In our study, ixekizumab treatment was associated with a reduction in the proportion of circulating IL-17–producing T cells (Th17 and Tc17 subsets), as assessed in peripheral blood mononuclear cells. IL-17A is a downstream cytokine in the pathogenesis of psoriasis, and thus biologics targeting IL-17A typically exhibit a relatively rapid onset of action. However, in patients with a higher proportion of Th17 and Tc17 cells, ixekizumab may not fully bind to and neutralize the IL-17A produced by these cells. Although ixekizumab did not alter the percentage of Treg cells, it improved the imbalance between Th17 cells and Treg cells to alleviate inflammation, which may contribute to its anti-inflammatory effects and represents one of the therapeutic advantages of ixekizumab.

Immune checkpoint molecules play a pivotal role in regulating immune responses, and therapies targeting these molecules have achieved significant efficacy in cancer treatment. Recently, there has been growing interest in exploring the relationship between immune checkpoints and various autoimmune diseases.

LAG-3, expressed on both activated and exhausted T cells, limits the suppressive function of Treg cells when chronic inflammation dominates (39, 59). Our study revealed a significant reduction in LAG-3 expression on circulating T cells after 24 weeks of ixekizumab treatment. Moreover, patients with higher baseline LAG-3 expression exhibited worse early responses, suggesting that elevated LAG-3 levels indicating an uncontrollable inflammatory response, delaying the onset of therapeutic effects. Previous studies reported that several inflammatory cytokines, such as IFN-γ and IL-6, can induce LAG-3 expression on human T cells (60, 61). Therefore, higher levels of LAG-3 expression may indicate more severe inflammation in patients. Additionally, it has been reported that CD4^+^ CD25^+^ LAG-3^+^ T Cells exhibit features of Th17 cells and are associated with disease activity in systemic lupus erythematosus (62). Coincidentally, a Phase I randomized study has shown that depletion of LAG-3^+^ T Cells can effectively treat psoriasis, with a return to baseline upon repletion of LAG-3^+^ T cells (41). Interestingly, we observed that after 12 weeks of treatment, LAG-3 expression on circulating T cells tended to increase, as reported in other studies from our team (43, 63). This may indicate that ixekizumab suppresses T cell activation in the early stages of treatment, while prolonged therapy may alleviate the disease by upregulating negative immune regulatory signals. As lesions resolve, LAG-3 expression is downregulated, and the mechanisms behind these dynamic changes warrant further investigation. Additionally, future studies should examine LAG-3 expression on Treg cells.

Previous studies have suggested that TIGIT expression triggers negative signaling events in T cells, and it is decreased on circulating CD4^+^ T cells, negatively correlating with PASI score (42, 64). In our study, we found that TIGIT expression tended to increase with disease duration, and baseline TIGIT expression on circulating CD4^+^ T cells negatively correlated with PASI score improvement at week 4. However, the exact role of TIGIT in psoriasis pathogenesis requires further exploration.

Alternative interpretations of our findings should be considered. Elevated baseline Th17 and Tc17 cell proportions may reflect a more inflammatory or refractory disease phenotype rather than representing ixekizumab-specific predictive markers. Higher IL-17–producing T cell levels could indicate a greater inflammatory burden, potentially requiring longer treatment duration to achieve clinical improvement and resulting in delayed early response. Similarly, increased LAG-3 expression may reflect chronic immune activation rather than a direct determinant of responsiveness to IL-17 blockade. Conversely, given the central role of the IL-23/IL-17 axis and the mechanism of action of ixekizumab, these parameters may also indicate pathway dependency. Comparative studies across biologics with different mechanisms of action are needed to clarify whether these markers are treatment-specific or reflective of overall disease severity.

For the first time, our study investigated the relationship between B7-H6 expression and psoriasis progression. We found that B7-H6 expression on CD4^+^ T cells tented to be negatively correlated with PASI scores, suggesting an anti-inflammatory role in psoriasis. After 24 weeks of ixekizumab treatment, B7-H6 expression on circulating CD4^+^ T cells was significantly reduced, potentially due to suppressed T cell responses. However, baseline B7-H6 level was not associated with clinical response at weeks 4, 12 or 24. Furthermore, the role of B7-H6-NKp30 axis and the crosstalk between activated T cells and NK cells warrants more investigation.

## 5 Conclusion

In conclusion, this study demonstrated that ixekizumab treatment was associated with a reduction in circulating Th17 and Tc17 cell proportions and decreased LAG-3 expression on CD4^+^ and CD8^+^ T cells in patients with plaque psoriasis. Baseline levels of Th17 and Tc17 cells, as well as LAG-3 expression, were associated with early clinical response at week 4. These findings suggest that these immune parameters may represent candidate exploratory biomarkers of early therapeutic response to ixekizumab.

## Data Availability

The original contributions presented in the study are included in the article/**Supplementary Material**. Further inquiries can be directed to the corresponding authors.
