# Supplementary material for: Baseline Th17/Tc17 and LAG-3 levels serve as candidate exploratory markers for early ixekizumab response in psoriasis
Source: Front Immunol. 2026 Mar 27;17:1653033. doi: 10.3389/fimmu.2026.1653033 (PMC13066243; doi:10.3389/fimmu.2026.1653033)
Supplement: Supplementary file 1 [file DataSheet1.pdf]

Supplementary Figures

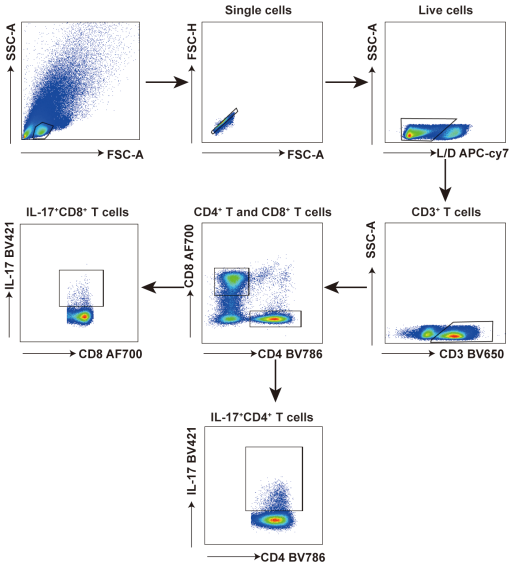

Figure S1. Flow cytometric gating strategies for Th17 cells

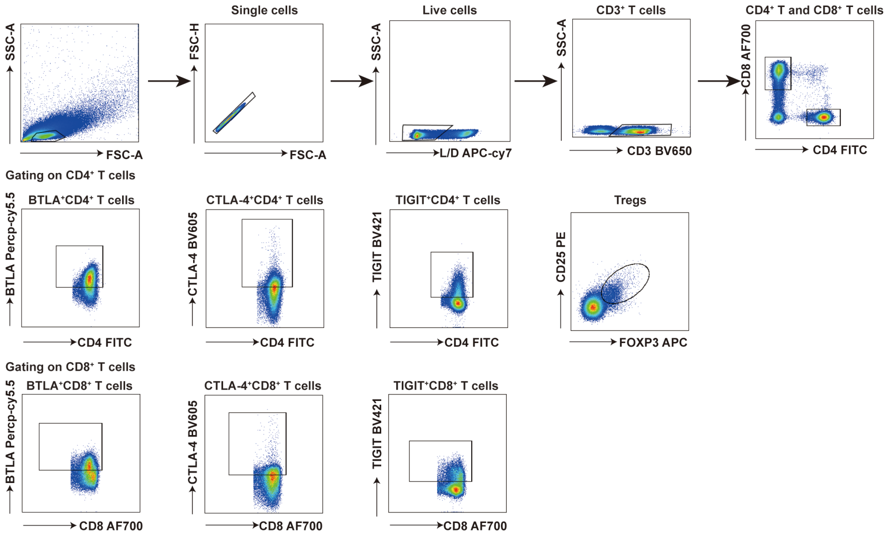

Figure S2. Flow cytometric gating strategies for CTLA-4, BTLA, TIGIT expression and Treg cells

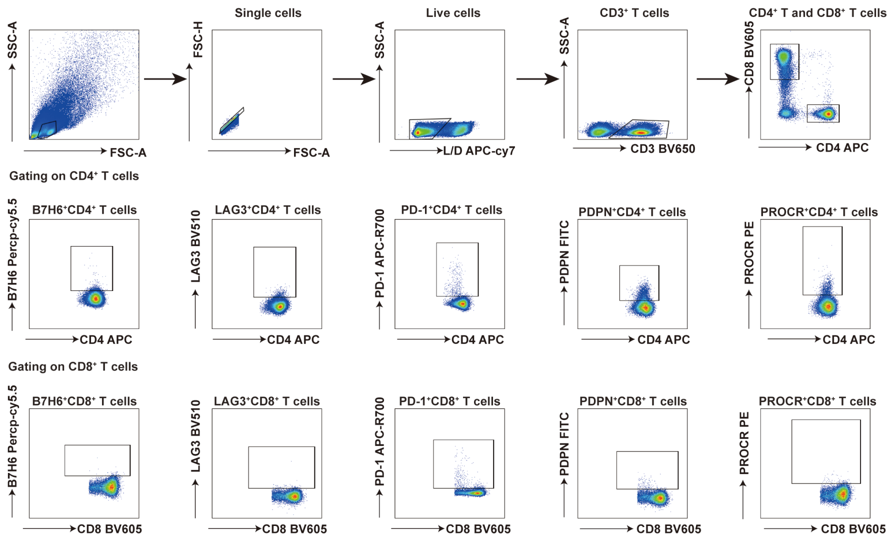

Figure S3. Flow cytometric gating strategies for B7H6, LAG-3, PD-1, PROCR, PDPN expression

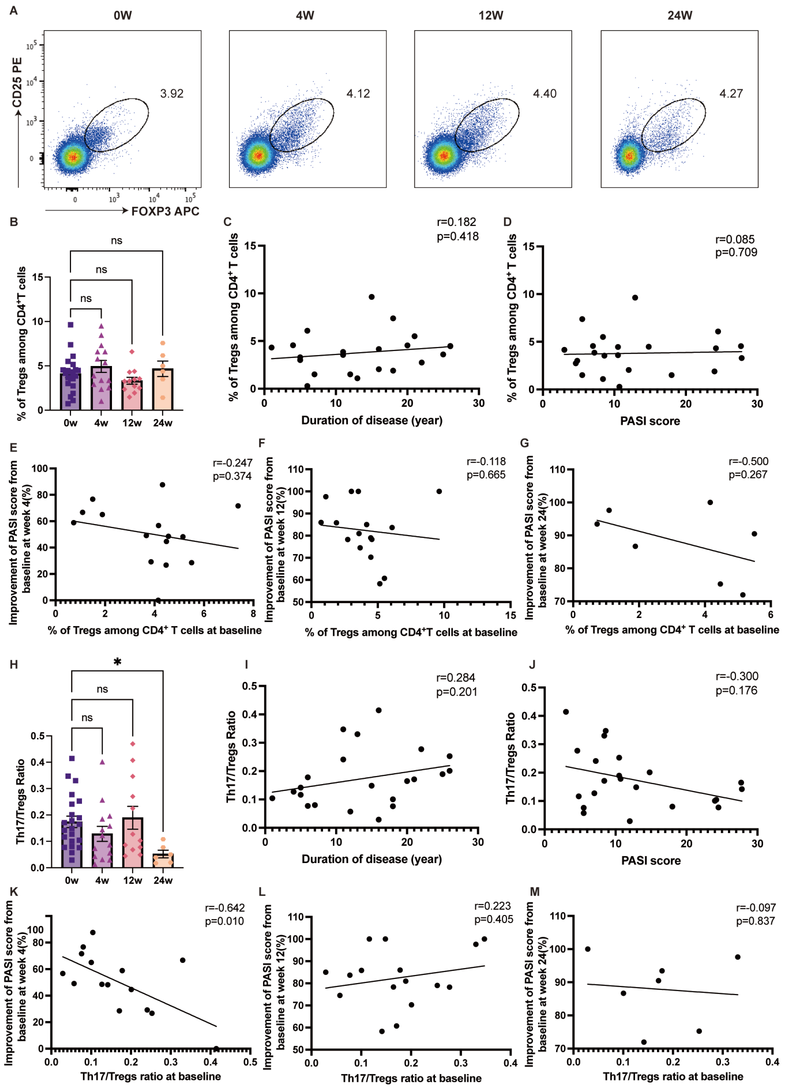

Figure S4. The circulating Treg cells and Th17/Treg ratio (A-B) The proportion of Treg cells in circulating CD4$^+$ T cells in psoriasis patients treated with ixekizumab at baseline (n=22), week 4(n=14), week 12 (n=12), and week 24 (n=6). (C) The correlation of Treg cell proportions in circulating CD4$^+$ T cells and the duration of disease of psoriasis patients (n = 22). (D) The correlation of Treg cell proportions in circulating CD4$^+$ T cells and PASI score (n=22). (E) The correlation of Treg cell proportions in circulating CD4$^+$ T cells and improvement of PASI score at week 4 (n=15). (F) The correlation of Treg cell proportions in circulating CD4$^+$ T cells and improvement of PASI score at week 12 (n=17). (G) The

correlation of Treg cell proportions in circulating CD4$^+$ T cells and improvement of PASI score at week 24 (n=7). (H) The Th17/Treg ratio in psoriasis patients treated with ixekizumab at baseline (n=22), week 4(n=14), week 12 (n=12), and week 24 (n=6). (I) The correlation of Th17/Treg ratio and the duration of disease of psoriasis patients (n = 22). (J) The correlation of Th17/Treg ratio and PASI score (n=22). (K) The correlation of the Th17/Treg ratio and improvement of PASI score at week 4 (n=15). (L) The correlation of the Th17/Treg ratio and improvement of PASI score at week 12 (n=17). (M) The correlation of the Th17/Treg ratio and improvement of PASI score at week 24 (n=7). *p < 0.05.

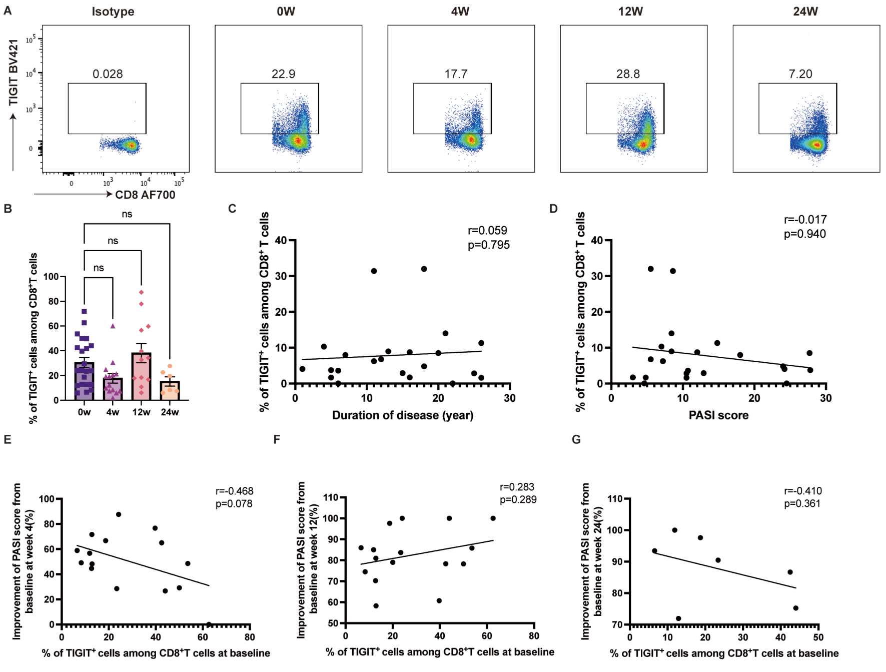

Figure S5. TIGIT expression on the circulating CD8$^+$ T cells (A-B) TIGIT expression on the circulating CD8$^+$ T cells in psoriasis patients treated with ixekizumab at baseline (n=22), week 4 (n=14), week 12 (n=12), and week 24 (n=6). (C) The correlation of TIGIT expression on the circulating CD8$^+$ T cells and the duration of disease of psoriasis patients (n = 22). (D)

The correlation of TIGIT expression in the circulating CD8$^+$ T cells and PASI score (n=22). (E) The correlation of the TIGIT expression and improvement of PASI score at week 4 (n=15). (F) The correlation of the TIGIT expression and improvement of PASI score at week 12 (n=17). (G) The correlation of the TIGIT expression and improvement of PASI score at week 24 (n=7).

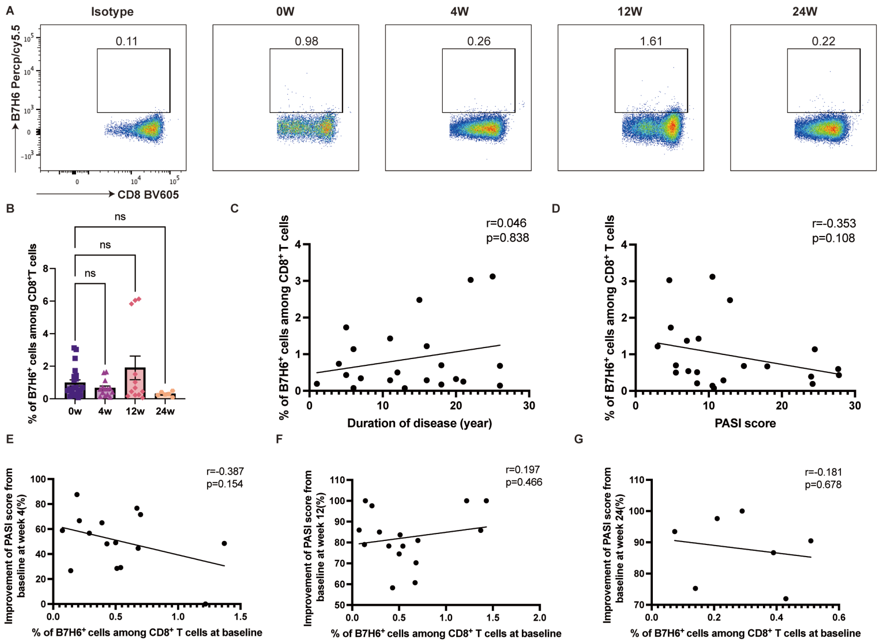

Figure S6. B7-H6 expression on the circulating CD8$^+$ T cells (A-B) B7-H6 expression on the circulating CD8$^+$ T cells in psoriasis patients treated with ixekizumab at baseline (n=22), week 4 (n=14), week 12 (n=12), and week 24 (n=6). (C) The correlation of B7-H6 expression on the circulating CD8$^+$ T cells and the duration of disease of psoriasis patients (n = 22). (D) The correlation of B7-H6 expression on the circulating CD8$^+$ T cells and PASI score (n=22). (E) The correlation of the B7-H6 expression and improvement of PASI score at week 4 (n=15). (F) The correlation of the B7-H6 expression and improvement of PASI score at week 12 (n=17). (G) The correlation of the B7-H6 expression and improvement of PASI score at

week 24 (n=7).

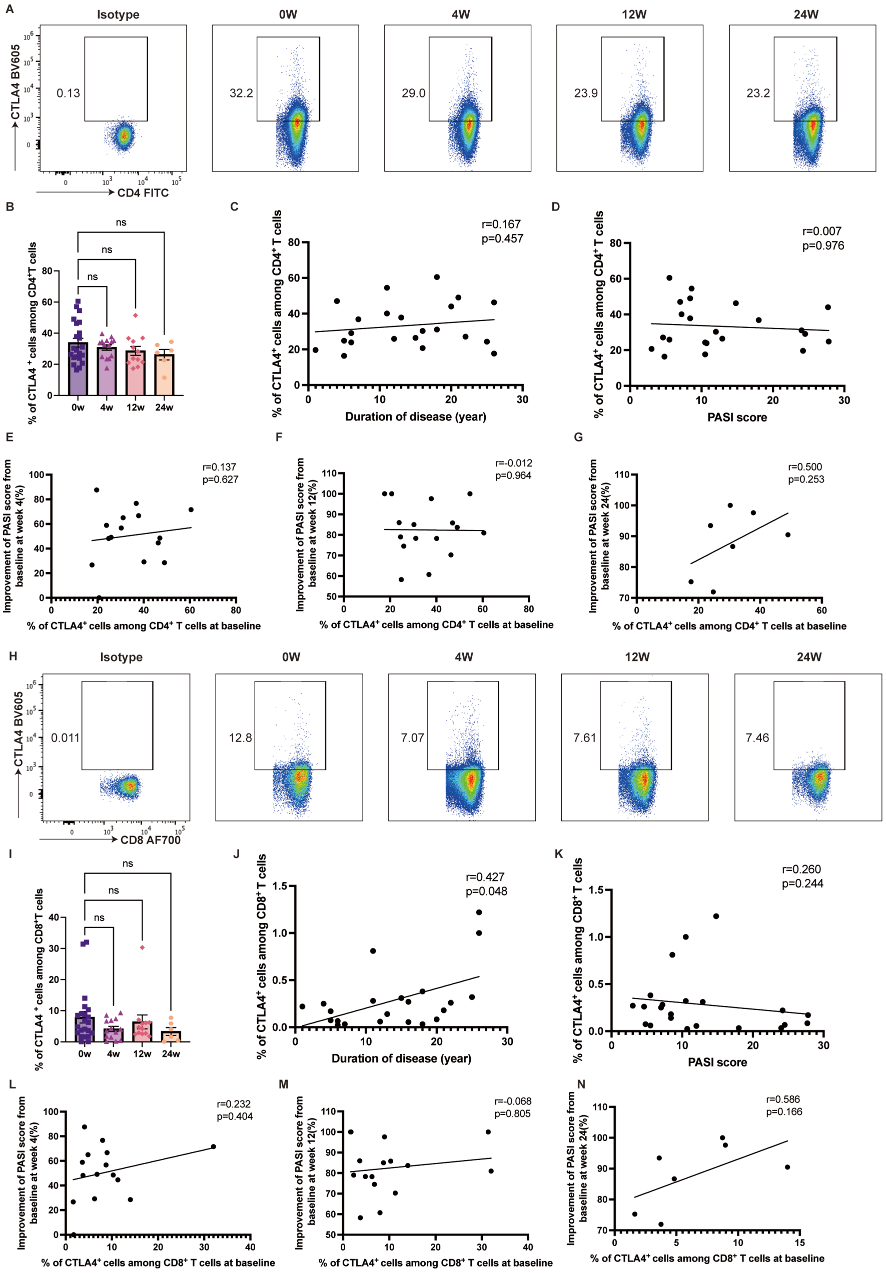

Figure S7. CTLA-4 expression on the circulating T cells (A-B) CTLA-4 expression on the circulating $CD4^+$ T cells in psoriasis patients treated with ixekizumab at baseline (n=22), week 4 (n=14), week 12 (n=12), and week 24 (n=6). (C) The correlation of CTLA-4 expression on the circulating $CD4^+$ T cells and the duration of disease of psoriasis patients (n = 22). (D) The correlation of CTLA-4 expression on the circulating $CD4^+$ T cells and PASI

score (n=22). (E) The correlation of the CTLA-4 expression on the circulating $CD4^+$ T cells and improvement of PASI score at week 4 (n=15). (F) The correlation of the CTLA-4 expression in the circulating $CD4^+$ T cells and improvement of PASI score at week 12 (n=17). (G) The correlation of the CTLA-4 expression on the circulating $CD4^+$ T cells and improvement of PASI score at week 24 (n=7). (H-I) CTLA-4 expression on the circulating $CD8^+$ T cells in psoriasis patients treated with ixekizumab at baseline (n=22), week 4 (n=14), week 12 (n=12), and week 24 (n=6). (J) The correlation of CTLA-4 expression on the circulating $CD8^+$ T cells and the duration of disease of psoriasis patients (n = 22). (K) The correlation of CTLA-4 expression on the circulating $CD8^+$ T cells and PASI score (n=22). (L) The correlation of the CTLA-4 expression and improvement of PASI score at week 4 (n=15). (M) The correlation of the CTLA-4 expression and improvement of PASI score at week 12 (n=17). (N) The correlation of the CTLA-4 expression and improvement of PASI score at week 24 (n=7).

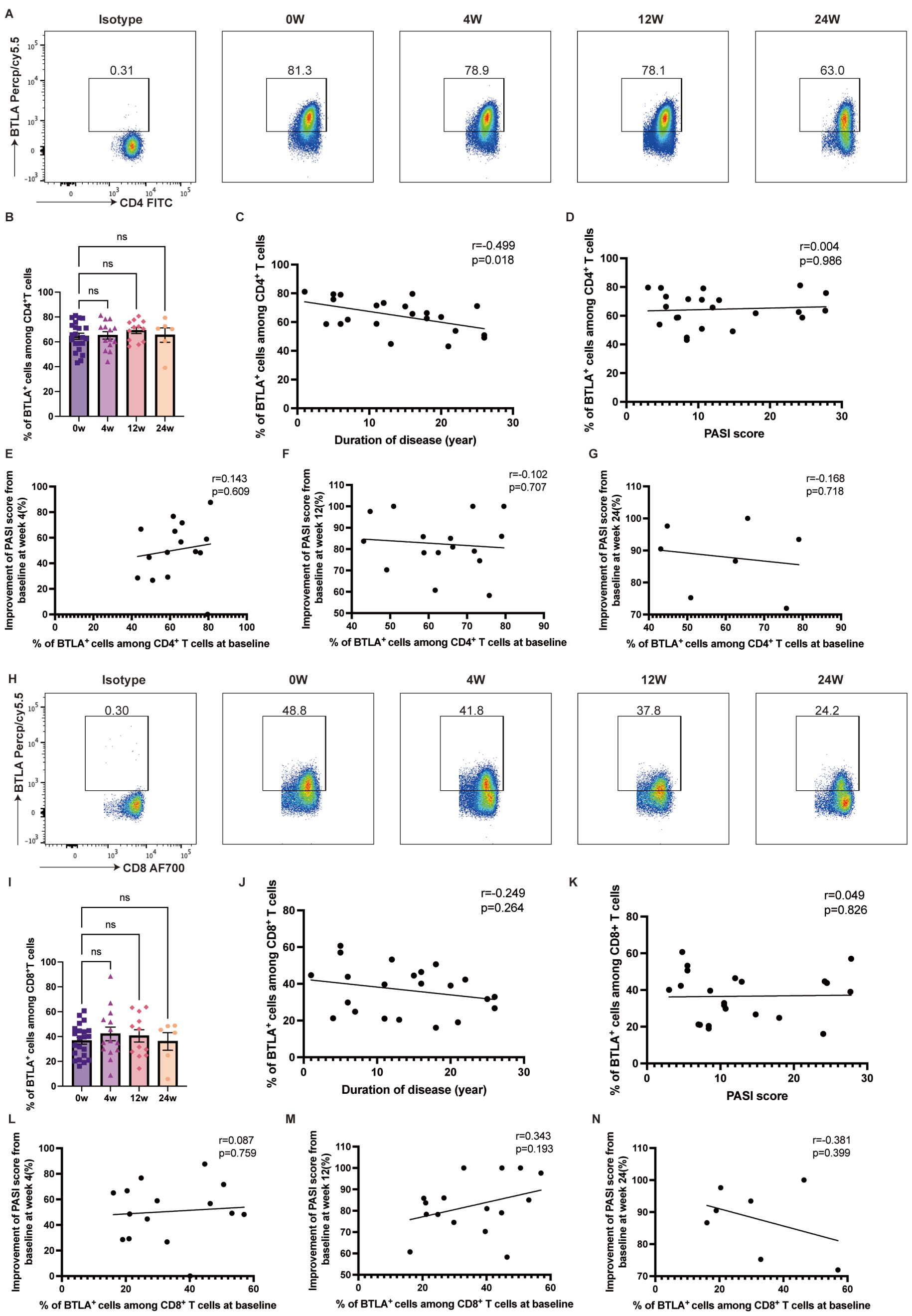

Figure S8. BTLA expression on the circulating T cells (A-B) BTLA expression on the circulating $CD4^+$ T cells in psoriasis patients treated with ixekizumab at baseline (n=22), week 4 (n=14), week 12 (n=12), and week 24 (n=6). (C) The correlation of BTLA expression on the circulating $CD4^+$ T cells and the duration of disease of psoriasis patients (n = 22). (D) The correlation of BTLA expression on the circulating $CD4^+$ T cells and PASI score (n=22). (E) The correlation of the BTLA expression on the circulating $CD4^+$ T cells and improvement

of PASI score at week 4 (n=15). (F) The correlation of the BTLA expression on the circulating $CD4^+$ T cells and improvement of PASI score at week 12 (n=17). (G) The correlation of the BTLA expression on the circulating $CD4^+$ T cells and improvement of PASI score at week 24 (n=7). (H-I) BTLA expression on the circulating $CD8^+$ T cells in psoriasis patients treated with ixekizumab at baseline (n=22), week 4 (n=14), week 12 (n=12), and week 24 (n=6). (J) The correlation of BTLA expression on the circulating $CD8^+$ T cells and the duration of disease of psoriasis patients (n = 22). (K) The correlation of BTLA expression on the circulating $CD8^+$ T cells and PASI score (n=22). (L) The correlation of the BTLA expression and improvement of PASI score at week 4 (n=15). (M) The correlation of the BTLA expression and improvement of PASI score at week 12 (n=17). (N) The correlation of the BTLA expression and improvement of PASI score at week 24 (n=7).

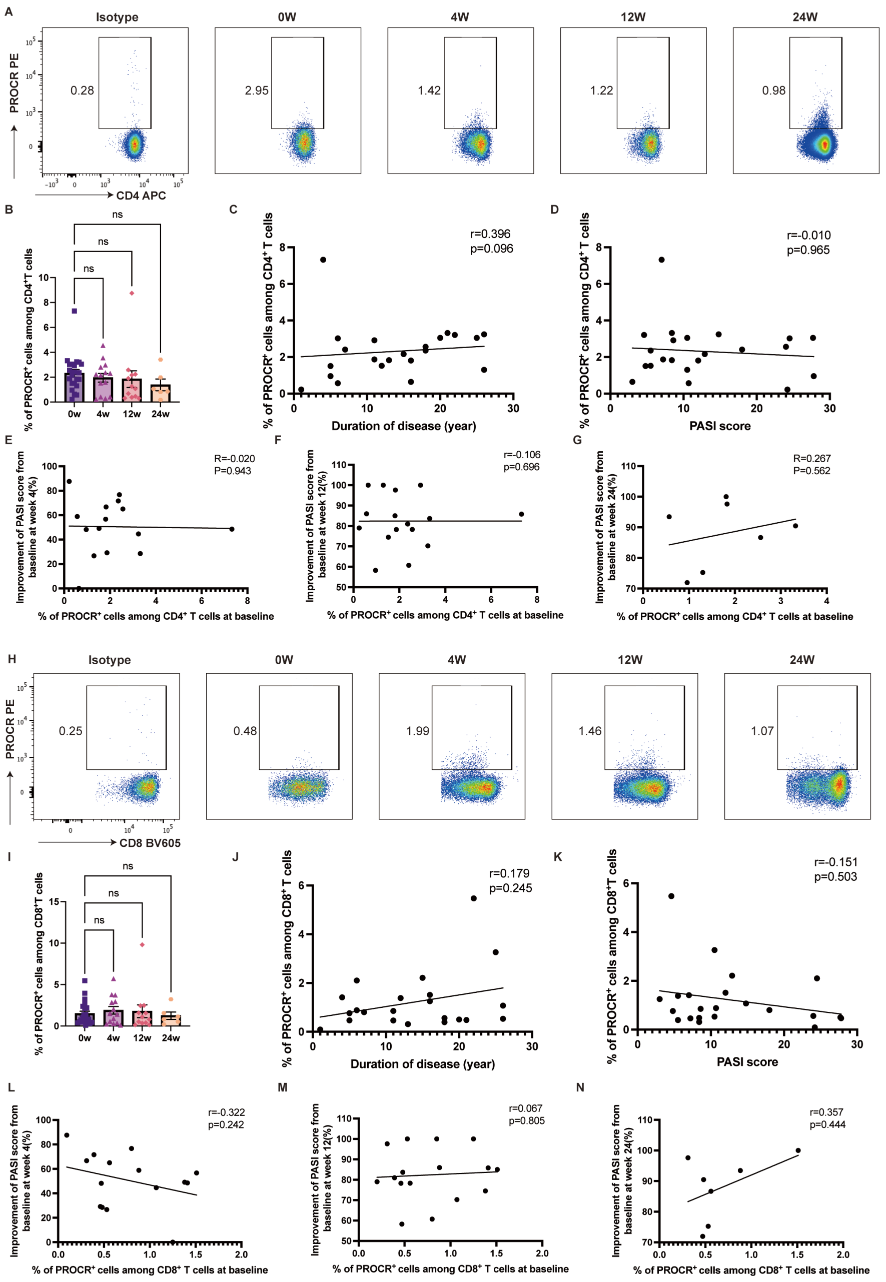

Figure S9. PROCR expression on the circulating T cells (A-B) PROCR expression on the circulating $CD4^+$ T cells in psoriasis patients treated with ixekizumab at baseline (n=22), week 4 (n=14), week 12 (n=12), and week 24 (n=6). (C) The correlation of PROCR expression on the circulating $CD4^+$ T cells and the duration of disease of psoriasis patients (n = 22). (D) The correlation of PROCR expression on the circulating $CD4^+$ T cells and PASI score (n=22). (E) The correlation of the PROCR expression on the circulating $CD4^+$ T cells and improvement of PASI score at week 4 (n=15). (F) The correlation of the PROCR

expression on the circulating $CD4^+$ T cells and improvement of PASI score at week 12 (n=17). (G) The correlation of the PROCR expression on the circulating $CD4^+$ T cells and improvement of PASI score at week 24 (n=7). (H-I) PROCR expression on the circulating $CD8^+$ T cells in psoriasis patients treated with ixekizumab at baseline (n=22), week 4 (n=14), week 12 (n=12), and week 24 (n=6). (J) The correlation of PROCR expression on the circulating $CD8^+$ T cells and the duration of disease of psoriasis patients (n = 22). (K) The correlation of PROCR expression on the circulating $CD8^+$ T cells and PASI score (n=22). (L) The correlation of the PROCR expression and improvement of PASI score at week 4 (n=15). (M) The correlation of the PROCR expression and improvement of PASI score at week 12 (n=17). (N) The correlation of the PROCR expression and improvement of PASI score at week 24 (n=7).

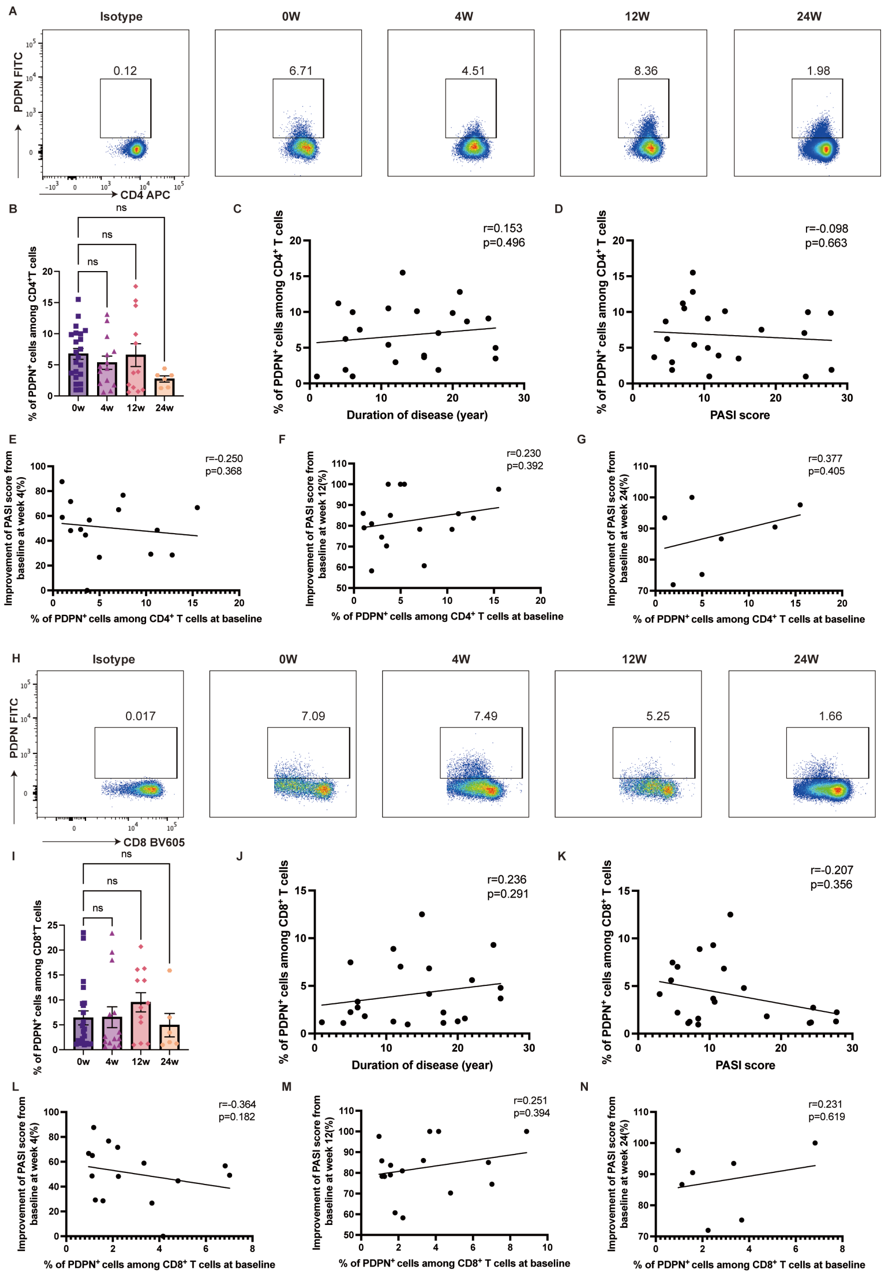

Figure S10. PDPN expression on the circulating T cells (A-B) PDPN expression on the circulating $CD4^+$ T cells in psoriasis patients treated with ixekizumab at baseline (n=22), week 4 (n=14), week 12 (n=12), and week 24 (n=6). (C) The correlation of PDPN expression on the circulating $CD4^+$ T cells and the duration of disease of psoriasis patients (n = 22). (D) The correlation of PDPN expression on the circulating $CD4^+$ T cells and PASI score (n=22). (E) The correlation of the PDPN expression on the circulating $CD4^+$ T cells and improvement of PASI score at week 4 (n=15). (F) The correlation of the PDPN expression on the

circulating $CD4^+$ T cells and improvement of PASI score at week 12 (n=17). (G) The correlation of the PDPN expression on the circulating $CD4^+$ T cells and improvement of PASI score at week 24 (n=7). (H-I) PDPN expression on the circulating $CD8^+$ T cells in psoriasis patients treated with ixekizumab at baseline (n=22), week 4 (n=14), week 12 (n=12), and week 24 (n=6). (J) The correlation of PDPN expression on the circulating $CD8^+$ T cells and the duration of disease of psoriasis patients (n = 22). (K) The correlation of PDPN expression on the circulating $CD8^+$ T cells and PASI score (n=22). (L) The correlation of the PDPN expression and improvement of PASI score at week 4 (n=15). (M) The correlation of the PDPN expression and improvement of PASI score at week 12 (n=17). (N) The correlation of the PDPN expression and improvement of PASI score at week 24 (n=7).

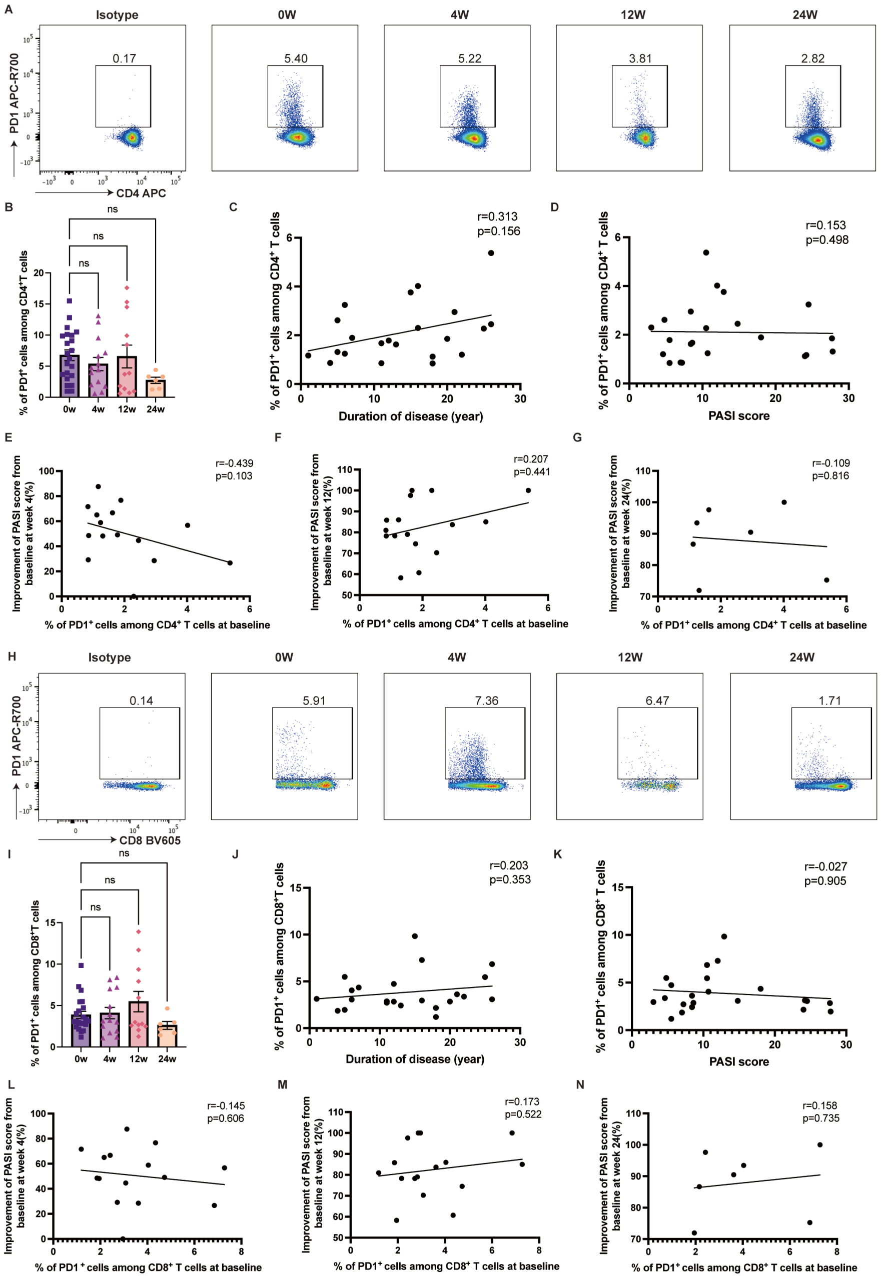

Figure S11. PD-1 expression on the circulating T cells (A-B) PD-1 expression on the circulating $CD4^+$ T cells in psoriasis patients treated with ixekizumab at baseline (n=22), week 4 (n=14), week 12 (n=12), and week 24 (n=6). (C) The correlation of PD-1 expression on the circulating $CD4^+$ T cells and the duration of disease of psoriasis patients (n = 22). (D) The correlation of PD-1 expression on the circulating $CD4^+$ T cells and PASI score (n=22). (E) The correlation of the PD-1 expression on the circulating $CD4^+$ T cells and improvement of PASI score at week 4 (n=15). (F) The correlation of the PD-1 expression on the circulating

CD4$^+$ T cells and improvement of PASI score at week 12 (n=17). (G) The correlation of the PD-1 expression on the circulating CD4$^+$ T cells and improvement of PASI score at week 24 (n=7). (H-I) PD-1 expression on the circulating CD8$^+$ T cells in psoriasis patients treated with ixekizumab at baseline (n=22), week 4 (n=14), week 12 (n=12), and week 24 (n=6). (J) The correlation of PD-1 expression on the circulating CD8$^+$ T cells and the duration of disease of psoriasis patients (n = 22). (K) The correlation of PD-1 expression in the circulating CD8$^+$ T cells and PASI score (n=22). (L) The correlation of the PD-1 expression and improvement of PASI score at week 4 (n=15). (M) The correlation of the PD-1 expression and improvement of PASI score at week 12 (n=17). (N) The correlation of the PD-1 expression and improvement of PASI score at week 24 (n=7).

Table. S1 Epidemiology of psoriasis patients

Characteristic	patients with psoriasis			P value
	total (n=22)	patients with PASI score at week 4 (n=15)	patients without PASI score at week 4 (n=7)	
Male, n (%)	18 (81.8)	14 (93.3)	4 (57.1)	0.077
Age, years, mean (SD)	37.2 (13.2)	37.7 (15.1)	36 (8.4)	0.781
PASI, mean (SD)	13.2 (7.6)	13.2 (7.5)	13.4 (9.2)	0.960
BSA, mean (SD)	17.4 (12.6)	17.4 (10.6)	17.3 (17.3)	0.666
DLQI, mean (SD)	12.7 (6.2)	12.9 (6.2)	12.3 (6.8)	0.830
PGA, mean (SD)	3.2 (0.8)	3.4 (0.7)	3.0 (0.8)	0.359
Duration of disease, years, mean (SD)	13.1 (7.7)	12.3 (7.8)	14.7 (8.0)	0.514

PASI: Psoriasis Area and Severity Index; BSA: body surface area; DLQI: Dermatology Life Quality Index; PGA: Physician's Global Assessment.
